# Functional Identification of the *Xanthomonas oryzae* pv. *oryzae* Type I-C CRISPR-Cas System and Its Potential in Gene Editing Application

**DOI:** 10.3389/fmicb.2021.686715

**Published:** 2021-08-12

**Authors:** Qibing Liu, Siwei Wang, Juying Long, Zhuoyue Chen, Bing Yang, Fei Lin

**Affiliations:** ^1^State Key Laboratory for Conservation and Utilization of Subtropical Agro-Bioresources/Key Laboratory of Natural Pesticide and Chemical Biology, Ministry of Education, South China Agricultural University, Guangzhou, China; ^2^Key Laboratory of Monitoring and Management of Plant Diseases and Insects, Ministry of Education, Nanjing Agricultural University, Nanjing, China; ^3^Division of Plant Sciences, Bond Life Sciences Center, University of Missouri, Columbia, MO, United States; ^4^Donald Danforth Plant Science Center, St. Louis, MO, United States

**Keywords:** type I CRISPR-Cas systems, *Xanthomonas oryzae* pv. *oryzae* (*Xoo*), CRISPR array, guide RNA, Cas protein, genome editing

## Abstract

The type I clustered regularly interspaced short palindromic repeats (CRISPR)/CRISPR-associated (Cas) system is one of five adaptive immune systems and exists widely in bacteria and archaea. In this study, we showed that *Xanthomonas oryzae* pv. *oryzae* (*Xoo*) possesses a functional CRISPR system by engineering constructs mimicking its CRISPR cassette. CRISPR array analysis showed that the TTC at the 5′-end of the target sequence is a functional protospacer-adjacent motif (PAM) of CRISPR. Guide RNA (gRNA) deletion analysis identified a minimum of 27-bp spacer that was required to ensure successful self-target killing in PXO99^A^ strain. Mutants with deletion of individual *Cas* genes were constructed to analyze the effects of Cas proteins on mature CRISPR RNA (crRNA), processing intermediates and DNA interference. Results showed that depleting each of the three genes, *cas5d*, *csd1*, and *csd2* inactivated the pre-crRNA processing, whereas inactivation of *cas3* impaired in processing pre-crRNA. Furthermore, the *Xoo* CRISPR/Cas system was functional in *Pseudomonas syringae* pv. *tomato*. Collectively, our results would contribute to the functional study of CRISPR/Cas system of *Xoo*, and also provide a new vision on the use of bacterial endogenous systems as a convenient tool for gene editing.

## Introduction

The CRISPRdb database^1^ predicts that approximately 87% of archaea and 45% of bacteria have the CRISPR-Cas system encoded in their genomes (Grissa et al., 2007). Even though the currently known CRISPR-Cas systems follow the same basic principle, they can be divided into 2 general classes (Class I and Class II), 6 types and 33 subtypes according to their composition (*Cas* gene content, repeat sequence and structure of CRISPR locus) (Makarova et al., 2020). The most widely used Class II systems rely on single-effecter proteins (a large protein structure domain), namely Cas9 (Type II), Cas12 and Cas14 (Type V) and Cas13 (Type VI). However, Class I systems that use multiple effector protein complexes to interfere with DNA/RNA include Cas3 (Type I), Cas10 (Type III), and dinG (Type IV) (Nethery and Barrangou, 2019).

Because the Class II CRISPR/Cas system only needs an effective protein that can interferes with DNA, the system has been widely engineered for gene editing in medicine, agriculture and biotechnology (Donohoue et al., 2018). However, although the widely used SpCas9 (derived from *Streptococcus pyogenes*) is of bacterial origin and genome editing based on Cas9 has been successful in several model organisms, its use in prokaryotes is rather limited (Chen et al., 2018). One of the reasons may be the cytotoxicity of Cas9 itself. Recently studies show that when Cas9 is introduced into *Corynebacterium glutamate* cells, colonies are not produced even in the absence of a guide RNA (gRNA) (Jiang and Doudna, 2017); overexpression of catalytically dead Cas9 in *E. coli* results in slow growth and abnormal morphology, suggesting that Cas9 also exhibits cellular toxicity through transient non-specific DNA binding of the genome (Cho et al., 2018). Therefore, we hope to explore other types of CRISPR systems that are different from the heterogenous Cas9 and implement additional re-editing strategies that have application potential. More notably, to date, the single effector Class II CRISPR-Cas system accounts for only 10% of all identified CRISPR-Cas systems. The remaining 90% belong to the diverse Class I system. Fifty percent of the identified systems belong to the type I CRISPR-Cas system of bacteria and archaea (Crawley et al., 2018). Therefore, we can reuse the endogenous type I CRISPR-Cas system in bacteria and archaea by synthesizing a mini CRISPR array. This will allow the endogenous Cascade-Cas3 complex to be redirected to the genome and reused for targeted killing, genome editing, or transcriptional control (Csorgo et al., 2020). In recent years, reusing these extensive and endogenously encoded CRISPR-Cas systems for “built-in” genome editing has become a simple, efficient and promising genetic manipulation strategy in prokaryotes (Cheng et al., 2017; Zhang et al., 2018).

*Xanthomonas oryzae* pv. *oryzae* (*Xoo*) is the pathogen responsible for rice bacterial blight, which causes serious loss of rice yield. Genome sequencing of three *Xoo* strains (MAFF 311018, KACC10331 and PXO99^A^) reveals that each strain contained a CRISPR/Cas system belonging to Class II of the type I-C clade (Salzberg et al., 2008). Sequence determination and phage infection assays in two strains (Xo21 and Xo604) suggested that CRISPR-mediated phage resistance also functions in *Xoo*. Bioinformatic analysis identified a conserved TTC sequence that is present next to the protospacer sequence of the *Xoo* bacteriophage but in the opposite orientation to *Streptococcus thermophilus* (Semenova et al., 2009). In this study, we confirmed TTC as the protospacer-adjacent motif (PAM) and determined the minimum length of the guide RNAs and *Cas* genes that were required for CRISPR function. Furthermore, the *Xoo* CRISPR-Cas system is functional in *Pseudomonas syringae* pv. *tomato*, indicating the possibility of utilizing the *Xoo* CRISPR-Cas system as a tool for gene editing in other organisms.

## Results

### Functional CRISPR/Cas System in *Xoo*

**FIGURE 1:**
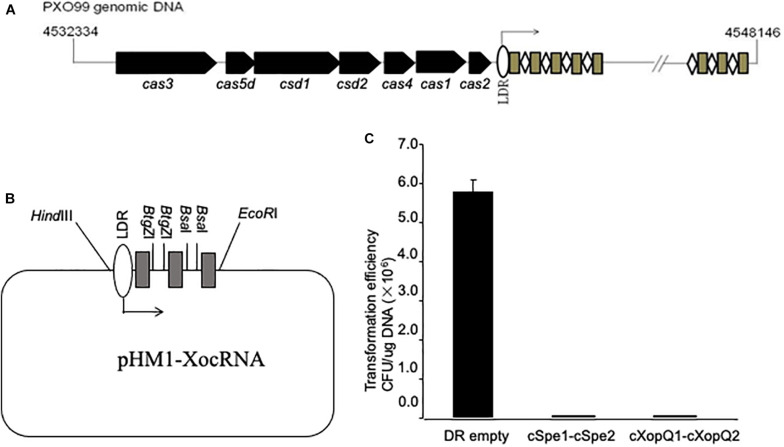
Engineering constructs mimicking *X. oryzae* pv. *oryzae* CRISPR cassette. **(A)** The *Xoo* CRISPR locus is schematically shown. *Cas* genes are identified and indicated by arrows. Repeats are indicated by grey rectangles, spacers by white rhombuses. The leader sequence (LDR) is located between the *cas2* gene and the CRISPR Cassette with the direction of transcription indicated. **(B)** Schematic diagram of the vector containing the CRISPR cassette mimicking that of *Xoo*. Sites of *Btg*Z I and *Bsa* I permit the insertion of two spacers for targeting sequences. **(C)** Plasmids containing spacers targeting spectinomycin resistance gene in the backbone of pHM1 or type III effector gene *xopQ* in *Xoo* were used for transformation assay of PXO99^A^. Plasmid pHM1-XocRNA DR empty was used as a control. Transformation efficiency was calculated as colony-forming units (CFU) per microgram of plasmid DNA. Error bars represent 5% SDs.

The genomic structure of the CRISPR system in the PXO99^A^ strain has been described (Figure 1A). To further facilitate the dissection of the CRISPR function, a construct mimicking the *Xoo* CRISPR cassette was designed and synthesized by using pUC57 as the backbone. The mimicking cassette contains an array of 90-bp TATA boxes, 100-bp leader sequences (LDRs), three “GTCGCGTCCTCACGGGCGCGTGGATTGAAAC” direct repeats (DRs) separated with two restriction sites for *Btg*ZI and *Bsa*I for inserting spacer sequences between DRs, ending with a 20-bp terminus (referred to herein as mini-CRISPR) (Figure 1B). We reasoned that the mimicking cassette containing the spacer sequences matching to the target sites in *Xoo* could trigger CRISPR interference in *Xoo* cells, transformation of mini-CRISPR construct into *Xoo* competent cells would result in the death of the cells. Plasmid pHM1-XocRNA-CSpec1-CSpec2 targeting the spectinomycin resistant gene was transferred into PXO99^A^ cells and plated on spectinomycin-containing medium. Only 8 spectinomycin resistant transformants were obtained, but lots of colonies were observed to form when using the control vector (Figure 1C). Similarly, when plasmid pHM1-XocRNA-XopQ1-XopQ2 targeting the type III effector gene (*xopQ*) was transferred into *Xoo* cells, only 4 spectinomycin resistant transformants were detected, while the control had lots of clones grown (Figure 1C). Altogether, the findings demonstrate that *Xoo* strain PXO99^A^ processes a functional CRISPR-Cas system and confirm that active CRISPR-Cas loci and their targets cannot coexist in the same cells.

### gRNA Requirements for CRISPR/Cas System Function

**FIGURE 2:**
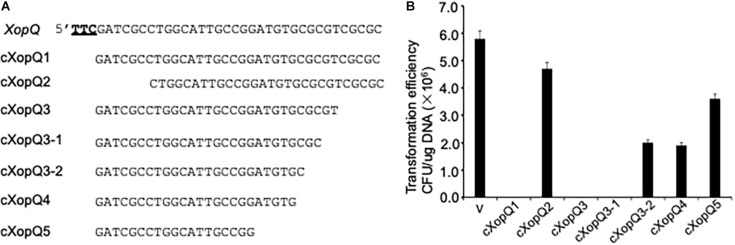
Detection of CRISPR function using various truncated forms of gRNA spacer sequences. **(A)** gRNA sequence targeting spectinomycin gene as a native version (cXopQ1). Various truncated spacer sequences of gRNA missing either their 5′ sequences or their 3′ sequences (cXopQ2, cXopQ3, cXopQ3–1, cXopQ3–2, cXopQ4, cXopQ5). The trinucleotide motif TTC, which is the conserved protospacer adjacent motif (PAM), is underlined in bold (XopQ). **(B)** Functional analysis of the truncated gRNA spacer sequences in PXO99^A^ cells. Transformation efficiency was measured as CFU per microgram of plasmid DNA. Error bars represent 5% SDs.

Bioinformatics analysis identified a trinucleotide motif, 5′-TTC-3′, present upstream of the most protospacers in *Xoo* phages. To confirm the prediction experimentally and determine the minimum length required for the functional gRNA, various truncated forms of gRNA lacking sequences corresponding to the native version (cXopQ1, Figure 2A) at their 5′ or 3′ ends were expressed in *Xoo* cells and then selected on the spectinomycin medium plates (Figure 2A). For the control plasmid (pHM1-XocRNA-DR empty), many colonies were grown, suggesting competency of bacterial cells and feasibility of transformation. When the plasmid pHM1-XocRNA-cXopQ1 was transferred into PXO99^A^ cells and plated on spectinomycin-containing medium, none of spectinomycin resistant transformants were obtained. However, deletion of five nucleotides (nt) from the 5′-end of gRNA, which juxtaposed to TTC (PAM), abolished the CRISPR function and resulted in the growth of many spectinomycin resistant colonies (pHM1-XocRNA-CXopQ2, Figure 2B). This result confirmed that the TTC motif is a genuine PAM that is essential for CRISPR function in *Xoo*. Truncation of the gRNA from the 3′-end showed that cXopQ3, which lacks five nucleotides (nt) from the 3′-end did not abolish the CRISPR activity. However, cXopQ4 and cXopQ5, which lack 10 or 15 nucleotides (nt) from the 3′-end, respectively, abolished the CRISPR activity (Figure 2B). Based on this finding, we further deleted two nucleotides (cXopQ3-1) and four nucleotides (cXopQ3-2) at the 3′-end of cXopQ3, respectively (Figure 2A). The cXopQ3-1 was still active, but the activity of cXopQ3-2 was lost (Figure 2B). We therefore conclude that a minimal stretch of 27-bp of the gRNA directly matching the sequence downstream of the TTC PAM is required for effective CRISPR function.

### Mutagenesis of *Cas* Genes

**FIGURE 3:**
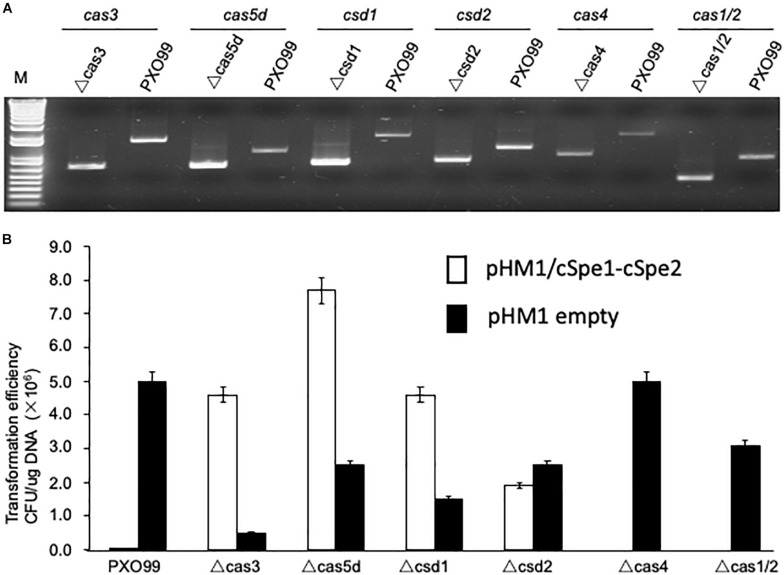
Mutagenesis of *Cas* genes in PXO99^A^ strain. **(A)** PCR verification of *Cas* gene knockout mutants derived from the PXO99^A^ strain. **(B)** Functional analysis of CRISPR in *Xoo* strains with the specified *Cas* gene inactivated. The non-targeting plasmid pHM1-XocRNA DR empty was used as a control.

To dissect the function of individual *Cas* genes in the *Xoo* CRISPR-Cas system, selection marker-free mutants were constructed for each of the 7 *Cas* genes. The mutants of individual genes were confirmed by comparing the size of the PCR product from the wild type and mutated alleles containing the deletion flanking fragments (Figure 3A). The 6 *Cas* mutants (*cas1* and *cas2* were deletion together), as well as the wild-type strain PXO99^A^, were transformed with plasmid pHM1-cSpe1-cSpe2 to test CRISPR function in each *Cas* gene deletion strain. The results showed that inactivation of four *Cas* genes, *cas3*, *cas5d*, *csd1*, and *csd2*, led to the observation of many spectinomycin resistant colonies on plates. However, deletion of the other three *Cas* genes (*cas4*, *cas1*, and *cas2*) did not increase the number of spectinomycin resistance transformants compared to the control stains (Figure 3B). The results showed that Cas3, Cas5d, Csd1, and Csd2 proteins were essentially required for the CRISPR function in *Xoo*, while Cas4, Cas1, and Cas2 proteins might be involved at other stages of the CRISPR-Cas interference.

**FIGURE 4:**
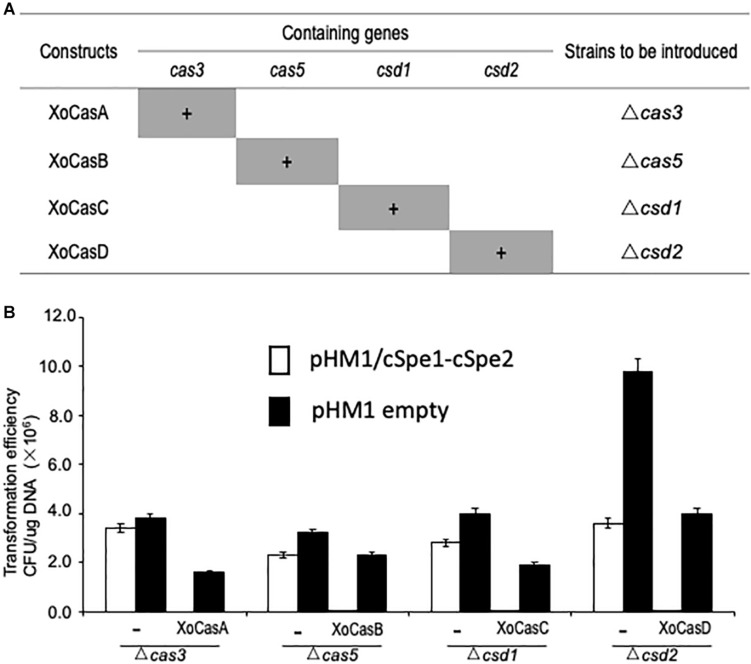
Restored CRISPR activity in *Cas* gene complementing strains. **(A)** Constructs used to transform *Cas* gene mutant strains. Genes present in each construction are shaded in grey. **(B)** CRISPR function was restored in the complementing strains. Competent cells of the *Cas* gene knockout strain with or without **(–)** corresponding complementary construct were used to test CRISPR activity by introducing gRNA targeting the spectinomycin resistance gene in the pHM1 vector.

Functional complementation tests were performed to confirm whether the loss of CRISPR function was due to the loss of the *Cas* gene in individual mutants. Four DNA fragments (XoCasA, XoCasB, XoCasC, and XoCasD) expressing different arrays of *Cas* genes were introduced into *Xoo* cells (Figure 4A). Cell death caused by CRISPR interference was observed when gRNA cSpe1-cSpe2 was delivered into the four complementation strains XoCasA, XoCasB, XoCasC, and XoCasD, suggesting the restoration of CRISPR function in each of *Cas* mutants (Figure 4B).

### Northern Analysis of Pre-crRNA Processing in *Cas* Gene Knockout Strains

**FIGURE 5:**
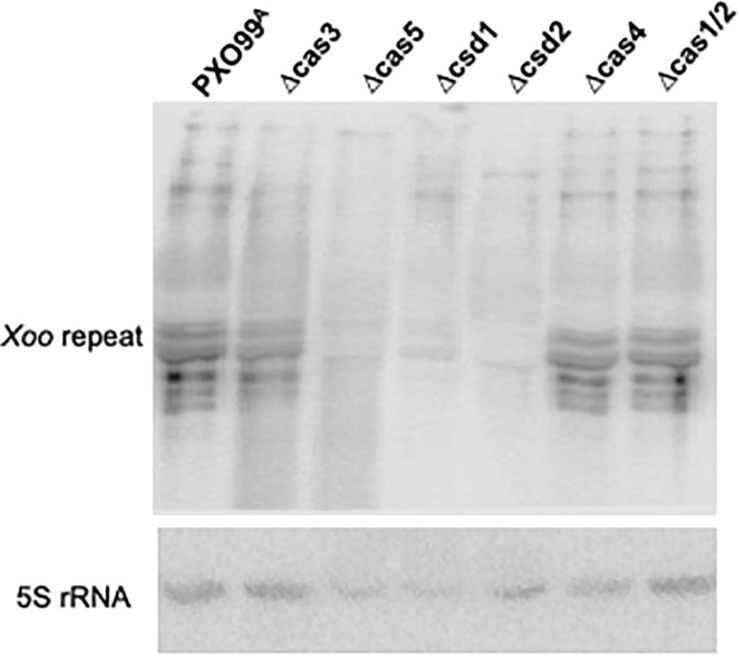
Functions of Cas proteins of the *Xoo* CRISPR system in pre-crRNA processing and PAM-dependent DNA silencing. Northern blot analysis of total RNAs from PXO99^A^ and its derived Cas gene knockout strains for pre-crRNA processing. Total RNA was probed using labeled oligonucleotides of the repeat sequences. Total RNA was hybridized with the 5S rRNA probe as a loading control.

Pre-crRNA processing in each of *Cas* gene knockout strains was tested by Northern blot analysis (Figure 5). Since neither intermediate nor mature crRNA was present in the mutant, deletion of each of the genes encoding the Cas5d, Csd1, and Csd2 proteins inactivated pre-crRNA processing. These results indicate that the Cas5d, Csd1, and Csd2 are essential for mature crRNA production in *Xoo* and that they are the three key enzymes responsible for pre-crRNA processing in this organism. However, the Northern blot patterns produced by the Δcas4 and Δcas1/2 strains were the same as those produced in the wild-type strains. The hybridization signal for the processed intermediates in the ΔCas3 strain was greatly reduced, while the crRNAs remained at very similar levels compared to the wild-type strain. These results indicate that Cas3 protein functions in stabilizing RNA intermediates of pre-crRNA, whereas Cas4 and Cas1/2 proteins do not appear to have any effect on the processing and maturation of pre-crRNA.

### *Xoo* CRISPR-Cas in *Pseudomonas syringae* pv. *Tomato*

**FIGURE 6:**
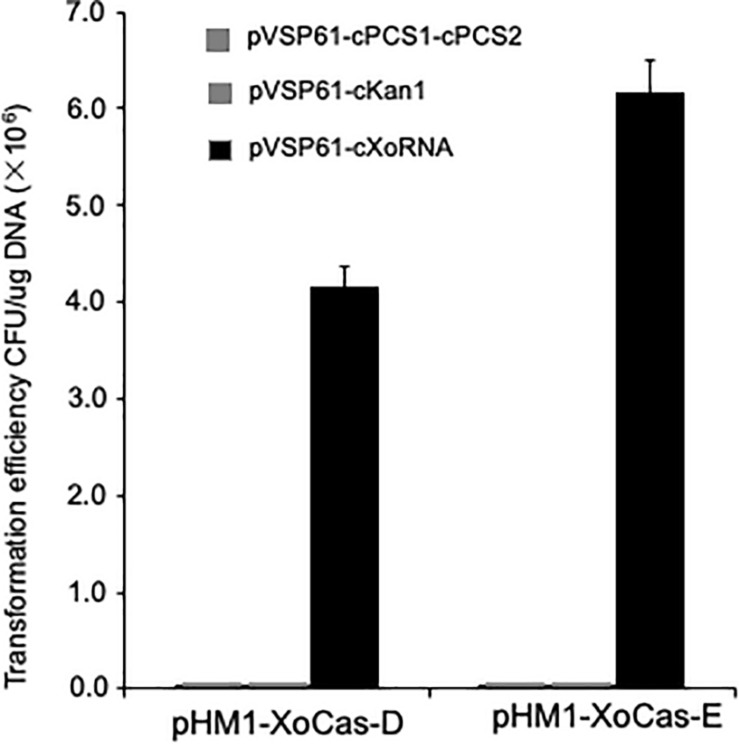
Detection *Xoo* CRISPR/Cas system function in *Pseudomonas syringae* pv. *tomato* strain DC3000. Plasmids (pVSP61-cKan1) contain gRNAs with spacers targeting kanamycin resistance gene or plasmids (pVSP61-cPCS1-cPCS2) contain the *PCS* gene in DC3000 in the backbone of pVSP61 vector were transferred to DC3000 cells containing plasmid expressing *Xoo Cas* genes. Plasmids (pVSP61-cXoRNA) contain gRNAs with spacers targeting *Xoo Cas* gene in the backbone of pVSP61 vector as a control. Plasmid pHM1-XoCas-D carries four *Cas* genes, *cas3*, *cas5d*, *csd1*, and *csd2*, which are required for CRISPR function from *Xoo*, while plasmid pHM1-XoCas-E contain all seven *Cas* genes from *Xoo*.

Since the CRISPR-Cas system has great potential for gene editing in different organisms, we expect that the *Xoo* CRISPR-Cas system also functions in another plant bacterial pathogen. *P. syringae* pv. *tomato* strain DC3000 was used to express the whole set of *Xoo Cas* genes (XoCasE) or the four essential *Cas* genes required for CRISPR function (XoCasD, without genes encoding Cas4, Csd1, and Csd2 proteins). gRNA targeting the kanamycin resistance gene in the pVSP61 vector backbone or the *PCS* (pyoverdine chromophore precursor synthetase) gene from *P. syringae* was used to examine the CRISPR function. Almost no colonies survived on the kanamycin medium when the gRNA was co-expressed with the *Xoo Cas* gene complex in DC3000 cells, while many clones grew from the control empty vector (Figure 6), suggesting that the *Xoo* CRISPR-Cas system is also functional in *P. syringae* pv. *tomato* and has potential to be engineered into a genome editing tool in bacteria.

## Discussion

Although the Type I-C system is the most common CRISPR-Cas system (Makarova et al., 2011), the functional characterization of CRISPR in *Xanthomonas* bacteria has not been reported in detail. Research has shown that the endogenous CRISPR-Cas system of *Xoo* can encode multiple Cas proteins, forming a multi-Cas-effectors system, namely CRISPR-related complex for antiviral defense.

Previous studies have shown that in the I-E CRISPR-Cas system of *E. coli*, the Cas1-Cas2 complex has a dual function. The complex not only needs to acquire the original protospacer DNA but is also responsible for the recognition of PAM in the process of protospacer capture (Wang et al., 2015). In this study, we showed that Cas1 and Cas2 proteins had no detectable effect on processing and maturation of pre-crRNA instead might involve in other processes of the CRISPR-Cas interference, suggesting that they indeed function in spacer acquisition. In addition to the participation of Cas1 and Cas2, endonucleases of the Cas4 family are one of the other accessory proteins aided in *de novo* protospacer acquisition. Cas4 was found to play an important role in the recognition of PAM and the determination of prespacer length during the adaptation of some archaeal models (Shiimori et al., 2018; Zhang et al., 2019). Cas4 overexpression inhibited the acquisition of protospacer fragments in *Sulfolobus islandicus* (Zhang et al., 2019), indicating that Cas4 might be used by viruses to inhibit CRISPR immunity as an anti-CRISPR factor (Nunez et al., 2016; Yoganand et al., 2017); Cas4 and Cas1 are similar to RecB- and AddB-type nucleic acid helicases (Heidrich and Vogel, 2013). In addition, fusion of Cas1 and Cas4 was found in Types I and III of several bacteria and archaea, indicating a correlation between the functions of the two proteins (Kieper et al., 2018; Rollie et al., 2018); The complex formed by Cas4 and Cas1 integrase in the endogenous Type I-C system of *Bacillus halogenated* efficiently cleaved the 3′ overhang of prespacers in a PAM-dependent manner, efficiently integrating the functional spacer moiety into the CRISPR array (Lee et al., 2018). These observations that depleting Cas4 or Cas1/2 neither blocked the processing crRNA nor degraded the DNA targets indicated that XoCas4 might indeed be functionally related to Cas1/Cas2.

Cas3 protein is the characteristic protein of the type I CRISPR-Cas system (Klompe et al., 2019). It is a multidomain nuclease-helicase, and it was reported that Cas3 has a dual role, functioning during CRISPR interference as well as during protospacer acquisition (Jiang et al., 2013). Depletion of XoCas3 completely block the degradation of the DNA target, but did not interfere with the processing of crRNA. This result suggests that XoCas3 indeed plays an important role during CRISPR interference, and whether it functions in protospacer acquisition or gene expression remains to be investigated.

The Cas5 protein is one of the most prevalent Cas protein families in the CRISPR-Cas system. Cas5 widely exists in Type I-A, Type I-B, and Type I-E systems, but only directly participates in the pre-RNA maturation process in the Type I-C system (Makarova et al., 2019). In *S. pyogenes*, Cas5d (now called Cas5c) (Reeks et al., 2013) specifically recognizes the hairpin structure in the pre-crRNA repeat of CRISPR Type I-C but does not recognize the unstructured pre-crRNA repeat of Type II-A. Previous studies showed that Cas5d not only had endoribonuclease activity independent of metal pair pre-crRNA processing, but also had hybrid DNA enzyme activity in the presence of divalent metal ions and non-specific double-stranded DNA binding affinity (Punetha et al., 2014). This indicates that Cas5 protein in the Type I-C system may play a role at multiple stages in CRISPR immunity (Koo et al., 2013). Furthermore, Cas5d assembles into an ∼400-kDa Cascade-like complex together with crRNA and the other two subtype-specific proteins Csd1 and Csd2, suggesting that Cas5d further participates in the crRNA-mediated DNA silencing step (van der Oost et al., 2014). These findings provide explanation for our finding that knockout of the *cas5d*, *csd1*, or *csd2* genes, resulted in blockage of CRISPR interference with the failure of pre-crRNA degradation.

Compared to the complicated gene editing tools in eukaryotic organisms, CRISPR Type I and Type III seem more manageable for genome editing in prokaryotes due to the efficient expression and assembly of Cas protein complexes in these organisms. Both Type I-A and Type III-B show the potential to be employed for testing CRISPR-based genome editing in *Sulfolobus islandicus* by enabling self-targeting (Selle and Barrangou, 2015). The endogenous CRISPR system widely present in bacteria and archaea has the potential to be engineered and used as a tool for genome editing, targeted killing or transcriptional control. Such a “built-in” genome editing strategy has been successfully developed in several bacterial and archaeal species (Zhang et al., 2018).

Our results suggest that for the application of the *Xoo* CRISPR-Cas system as a gene editing tool in eukaryotes, at least four Cas proteins, Cas3, Cas5d, Csd1, and Csd2, need to be heterologously expressed in gene editing organisms. In addition, a stretch of at least 27-bp of the gRNA directly matching the downstream protospacer of the TTC PAM is required for efficient CRISPR function. Transfer of an artificial mini-CRISPR array to *Xoo* cells activates self-targeting, highlighting the potential for genome editing applications using an endogenously active system. However, there are potential biological hurdles. Genetic analysis of transformants recovered following self-targeting of the plasmid–encoded protospacer by the chromosomally encoded Cas machinery is needed to effectively preclude self-targeting. We found that *Xoo* cells survived from self-targeting on the antibiotic medium still contained the intact target DNA sequences, even though we combined gRNA and editing DNA template in one plasmid or increased the concentration of antibiotic for selection. The inefficiently cleared wild-type colonies from the population might be partially due to the low capacity of bacterial DNA repair mechanisms to cope with Cas cleavage or the low efficiency of gRNA delivery into *Xoo* cells. Thus, increasing transformation efficiency for delivery of editing templates and expression vectors is an experimental requirement for applying the *Xoo* CRISPR-Cas system as a gene editing tool in prokaryotic organisms. However, considering that thousands of colonies that grew on the selection medium contained empty vector, the explanation of the low transformation rate does not seem to hold. The mechanism underlying the high rate of anti-self-targeting needs to be further studied.

## Conclusion

Taken together, our study confirmed that *Xoo* possesses a functional CRISPR system. Various truncated forms of gRNA spacer sequences identified a stretch of at least 27-bp matching directly the downstream sequence of the TTC PAM as part of gRNA being required for efficient CRISPR function. Furthermore, depleting each of the three genes, *cas5d*, *csd1*, and *csd2* inactivates pre-crRNA processing, whereas inactivating *cas3* impairs processing pre-crRNA. The *Xoo* CRISPR/Cas system is functional in *P. syringae* indicating the possibility of utilizing the *Xoo* CRISPR/Cas system as a tool for gene editing in other organisms. Analysis of the *Xoo* CRISPR/Cas system provides new insights for the utilization of bacterial endogenous systems in this study.

## Materials and Methods

### Bacterial Strains, Plasmids, Culture Conditions

The strains of *E. coli*, *Xoo*, and *P. st* and the plasmids used in this study are described in Supplementary Table 1. *E. coli* was grown on LB medium at 37°C while *Xoo* strains were cultured on tryptone sucrose (TS) medium at 28°C. Strain of *Pst* DC3000 was incubated at 28°C in King’s B medium. The media were supplemented with the antibiotics, ampicillin (100 μg/ml), kanamycin (50 μg/ml), and spectinomycin (100 μg/ml) depending on the strains used. *In vivo* CRISPR-Cas activity was measured by conversion efficiency calculated as colony forming units (CFU) per μg of DNA after bacterial transformation.

### Constructs Expressing *Xoo* CRISPR Cassette

The sequence of the CRISPR Cassette in PXO99^A^ was used as a template for mimicking. An artificial mini-CRISPR array was synthesized by GenScript^2^ (Figure 1 and Supplementary Table 1). This CRISPR array was cloned into the intermediate vector pENTR4 at *Hin*dIII and *Eco*RI sites, resulting in empty pENTR4-DR (Supplementary Table 1). gRNAs were generated by annealing the corresponding complementary oligonucleotides (Supplementary Table 1) and sequentially cloning into the pENTR4-DR empty vector predigested with *Btg*ZI and *Bsa*I. Finally, the *Xoo* CRISPR Cassette was inserted into the broad host range vector pHM1 or pVSP61 at *Hin*dIII and *Eco*RI sites and subjected to *in vivo* CRISPR activity assay.

### Constructs of *Xoo Cas* Gene Deletion Mutations and Their Complementation

Deletion mutations of each *Cas* gene were constructed in *Xoo* strain PXO99^A^ using homologous recombination and the suicide vector pKMS1. For individual *Cas* genes, two fragments flanking the left and right of the gene were PCR-amplified using the genomic DNA of strain PXO99^A^ as template and the primer pairs listed in Supplementary Table 1. The PCR fragments were cloned into pGEM-T (Promega), then subcloned into the vector pKMS1 at the sites of *Bam*HI and *Hin*dIII, introduced into PXO99^A^ by electroporation and screened for deletion mutants essentially using the method as described previously (Zou et al., 2011). For the generation of complementary strains for each *Cas* gene deletion, PCR fragments covering the *Cas* genes were produced and cloned into the pHM1 vector at the restriction sites *Bam*HI and *Hin*dIII.

### Northern Blot Analysis

For Northern blot analysis, total small RNAs were prepared from the PXO99^A^ wild-type strain, its knockouts (Δ) of individual *Cas* genes by a miRNeasy kit (Qiagen, Beijing, China). Ten micrograms of total RNA extracted from each sample was separated on 12% PAGE gels and then transferred to a Hybond^TM^-N^+^ positively charged nylon membrane (Roche Diagnostics). The 5S rRNA probe was used as an internal control with oligoprobes, whereas the *Xoo* repeats was probed using a mixture of P^32^-labeled DNA antisense oligoprobes. Radiolabeled signals were observed by a Storm 860 Molecular Imager (Molecular Dynamics, Sunnyvale, CA).

## Data Availability Statement

The original contributions presented in the study are included in the article/Supplementary Material, further inquiries can be directed to the corresponding author/s.

## Author Contributions

BY and FL designed the research and modified the first draft of the manuscript. QBL, SWW, and FL performed the experiments and analysis data. JYL constructed the *Cas* gene deletion strains. QBL wrote the first draft of the manuscript. All authors read and approved the final manuscript.

## Conflict of Interest

The authors declare that the research was conducted in the absence of any commercial or financial relationships that could be construed as a potential conflict of interest.

## Publisher’s Note

All claims expressed in this article are solely those of the authors and do not necessarily represent those of their affiliated organizations, or those of the publisher, the editors and the reviewers. Any product that may be evaluated in this article, or claim that may be made by its manufacturer, is not guaranteed or endorsed by the publisher.

## Footnotes

1
https://crispr.i2bc.paris-saclay.fr/

2
https://www.genscript.com/
