# Supplementary material for: Functional Identification of the Xanthomonas oryzae pv. oryzae Type I-C CRISPR-Cas System and Its Potential in Gene Editing Application
Source: Front Microbiol. 2021 Aug 12;12:686715. doi: 10.3389/fmicb.2021.686715 (PMC8406640; doi:10.3389/fmicb.2021.686715)
Supplement: Supplementary file 1 [file Table_1.DOCX]

**Table S1 Primers used in present study.**

| Primer name | Primer sequence (5'–3') | Use |
| --- | --- | --- |
| XooCas-F1 | gatcggaTCCTGCTCAAGGATGACTCGCGT | Complementation test |
| XooCas-R1 | gcatgaatTCAATCACTTGGAAATTTCGCGAG |  |
| XooCas-F2 | gatcgaattcTACTGACTTATGAGTAACGCAC |  |
| XooCas-R2 | ATCCGGGTGGTCGACAACGCTTC |  |
|  |  |  |
| Cas3-F1 | atgcggatccTACACAGAAAAATTTCAGGCACG | Cas gene deletion template |
| Cas3-R1 | GTTGACGATGCACAGCACTTGTGAAAGCCGCTCATGCAGAGATG |  |
| Cas3-F2 | ACAAGTGCTGTGCATCGTCAAC |  |
| Cas3-R2 | cgatgtcgacTTCGTCGTCCAGTAGGTTGTC |  |
| Cas5-F1 | atgcggatccTACTCGTCAGAAGTGATGTTATG |  |
| Cas5-R1 | AGGACACCCGCTCCACCTTCATTTC |  |
| Cas5-F2 | ATGAAGGTGGAGCGGGTGTCCTGCTGGATGCTGCACGACATCGAC |  |
| Cas5-R2 | gcatgtcgacTCCATCTTTCGATGAATGCCA |  |
| Csd1-F1 | atcgagatctGATGCTGCACGACATCGACTTC |  |
| Csd1-R1 | GCCAATCTTCTCCCGACTGTAG |  |
| Csd1-F2 | CTACAGTCGGGAGAAGATTGGCCTCGATACAGGCAATACCGATC |  |
| Csd1-R2 | gcatgtcgaCGACGTAGTTACGGATCTTGC |  |
| Csd2-F1 | agtcggatccATCCAGGAACAAGGCCGCTTC |  |
| Csd2-R1 | GATGACGTCGAACAGGTAGAC |  |
| Csd2-F2 | GTCTACCTGTTCGACGTCATCGCCGACTACCGGGTCACGATC |  |
| Csd2-R2 | gcatgtcgaCGCACAACTGGACCTCATCG |  |
| Cas4-F1 | atgcggatccGCCGACTACCGGGTCACGATC |  |
| Cas4-R1 | CCACTGCTGCTCCACATGGATTAG |  |
| Cas4-F2 | CTAATCCATGTGGAGCAGCAGTGGAGCGTtGACCAGTGGGTCCGC |  |
| Cas4-R2 | gcatgtcgacGAGCCGCTTCAAAGAGTGCTG |  |

| Primer name | Primer sequence (5'–3') | Use |
| --- | --- | --- |
| Cas1/2-F1 | gatcggatccATGCTGCACGACAGCGACACG | Cas gene deletion template |
| Cas1/2-R1 | GGCATACAGTGTGTTGAGTTG |  |
| Cas1/2-F2 | CAACTCAACACACTGTATGCCGTAGAGCACGTCGGTGCCAAG |  |
| Cas1/2-R2 | gcatctcgagACCCCGCCAGCATAAGTGCTTG |  |
|  |  |  |
| cSpe-F1 | aaacGCGCTTAGCTGGATAACGCCACGGAATGATGTCG | gRNA annealing |
| cSpe-R1 | cgacCGACATCATTCCGTGGCGTTATCCAGCTAAGCGC |  |
| cSpe-F2 | aaacGAGATGGCGCTCGATGACGCCAACTACCTCTGAT |  |
| cSpe-R2 | cgacATCAGAGGTAGTTGGCGTCATCGAGCGCCATCTC |  |
| cXopQ-F1 | aaacGATCGCCTGGCATTGCCGGATGTGCGCGTCGCGC |  |
| cXopQ-R1 | cgacGCGCGACGCGCACATCCGGCAATGCCAGGCGATC |  |
| cXopQ-F2 | aaacTCGCCTGGCATTGCCGGATGTGCGCGTCGCGC |  |
| cXopQ-R2 | cgacGCGCGACGCGCACATCCGGCAATGCCAGGCGA |  |
| cXopQ-F3 | aaacGATCGCCTGGCATTGCCGGATGTGCGCGT |  |
| cXopQ-R3 | cgacACGCGCACATCCGGCAATGCCAGGCGATC |  |
| cXopQ-F6 | aaacGATCGCCTGGCATTGCCGGATGTGCGC |  |
| cXopQ-R6 | cgacGCGCACATCCGGCAATGCCAGGCGATC |  |
| cXopQ-F7 | aaacGATCGCCTGGCATTGCCGGATGTGC |  |
| cXopQ-R7 | cgacGCACATCCGGCAATGCCAGGCGATC |  |
| cXopQ-F4 | aaacGATCGCCTGGCATTGCCGGATGTG |  |
| cXopQ-R4 | CACATCCGGCAATGCCAGGCGATC |  |
| cXopQ-F5 | aaacGATCGCCTGGCATTGCCGG |  |
| cXopQ-R5 | CCGGCAATGCCAGGCGATC |  |
| cKan-F1 | aaacCTTGCGCAGCTGTGCTCGACGTTGTCACTGAAGC |  |
| cKan-R1 | cgacGCTTCAGTGACAACGTCGAGCACAGCTGCGCAAG |  |
